# The Public Health: Interviews with Local Authorities

**Published:** 1923-01

**Authors:** 


### THE PUBLIC HEALTH.

### INTERVIEWS WITH LOCAL AUTHORITIES.

### VI.—PORTSMOUTH.—SAFETY FIRST.

SPECIAL interest attaches to our interview with the Public Health Authority at Portsmouth in view of the fact that the Chairman, Mr. Childe, is an eminent surgeon who is President-elect of the British Medical Association. The Association propose to have their annual conference at Portsmouth this year, and there is a fitness in their selection, considered from the point of view of the vital statistics of the town, which are exceptionally good, the death rate of 11.2 per 1,000 being the third lowest in the group of large towns. As we stepped out of the train at Portsmouth we could not fail to be impressed by the majestic Town Hall, which is immediately outside the station. This fine building should be in itself an inspiration to those entrusted with the care of the health of the citizens of Portsmouth, while the very noble War Memorial, which immediately adjoins the Town Hall, should not fail to serve as a constant reminder and stimulus to those in authority that no better service could be rendered to the memory of Portsmouth's fallen sons than the constant sympathetic care of those who remain to carry on the traditions of this great naval town.

#### The Sailor and the Child.

An indication—if one were needed—of the character of Portsmouth's population, and of the responsibilities of the Health Committee, was furnished to us, quite simply, by the sight of a sailor pushing a pram; the sailor, a reminder of the ships and barracks and dockyard establishments which are Portsmouth's *raison d'être*, and the human bundle in the pram, the first object of the concern of any Health Committee which knows its job. Let us appreciatively record that in the year 1920 Portsmouth had the lowest infantile mortality rate ever reached in any of the twenty large towns, a rate of 60 per 1,000, to which the rate in 1921 (63 per 1,000) closely approximated; these figures are indeed significant when compared with the rate of nearly 160 of only twenty years ago.

#### Safety First.

The population of the borough has increased during the last twenty years at a considerably higher rate than in any of the other large towns, and at present numbers just under a quarter of a million persons. In pursuance of the principle of "safety first," which seems to be the keynote of the health administration in Portsmouth, Dr. Fraser has issued a little book of notes on infectious diseases for the use of teachers in the public elementary schools. We have seen elsewhere that parents and others are still inclined to take too light-heartedly attacks of such infantile complaints as measles and whooping-cough, notwithstanding that these have proved within recent years to be the most fatal of the infectious diseases amongst children. A book for the guidance of school teachers is therefore very valuable. Everyone interested in school hygiene appreciates the numerous opportunities which occur to teachers for assisting in the control of infectious diseases; in fact, no machinery for the purpose can be regarded as satisfactory in which the hearty co-operation of school teachers does not form an integral part. But, however anxious teachers may be to assist, the effectiveness of their co-operation must, in the first place, depend on their possessing an intimate knowledge of the most usual signs and symptoms of infectious disease.

Another valuable piece of publicity is the special leaflet issued to the public on the subject of cancer, upon which Mr. Childe is a great authority. It is pointed out that many persons die from cancer whose lives could be saved if they acted upon the advice therein given.

#### Dustbins and Disinfectants.

In Portsmouth the Health Authorities are alive to such comparatively uninspiring subjects as dustbins and disinfectants. As regards the former, they have in fact taken powers under the Corporation Act of 1920 to require the occupiers of premises to provide suitable galvanised iron dustbins with proper covers. With regard to disinfectants, the Corporation have their own disinfecting station, in which they manufacture electrolysed sea-water disinfectant. They distribute some 13,000 gallons yearly of this valuable disinfectant, which is produced at a very cheap rate; about half of this quantity is supplied to the public swimming-baths, schools and other institutions.

#### Maternity and Child Welfare.

Reference has been made above to the great improvement in the infant mortality rate. Dr. Fraser says that there is no doubt that the Midwives Act of 1902 has effected a very great improvement in the practice of midwifery work amongst the working classes, and it has been not the least factor in the reduction of the infant mortality rate. The Municipal Maternity Hospital opened in 1920 had 236 patients in 1921 admitted to its bright and lofty wards. The hospital is under the medical superintendence of Dr. Mabel Ross who has charge of the Child Welfare Centres, and it has been recognised as a qualifying training centre for midwives by the Central Midwives' Board. The fact that there were nearly 20,000 attendances at the Child Welfare centres during the year 1921, taken in conjunction with the record of 14,000 visits paid by the health visitors, is a sufficient indication of the thoroughness of the work which is being directed to the saving of child life in the Borough. If Charles Dickens, who was born in the Commercial Road, Portsmouth, could come again to his native place, he would transform these statistics in such wise that those who read their tale would do so with a very full heart. It is a pity to have to strike one jarring note, but the Medical Officer of Health rightly says that it is disquieting to see that the number of children who escaped vaccination in 1921 on the ground of the conscientious objections of their parents was so high as 1,300, or about one-fifth of the registered births in that year.

THE PREVENTION OF VENEREAL DISEASE.

Perhaps the most striking feature of Public Health administration in Portsmouth in recent years is the courageous stand made by the Medical Officer in the report presented to his Committee early in 1920, demanding an active educational campaign in the prevention of venereal disease. He pointed out that the great tragedy in connection with venereal diseases is the host of innocent women and child victims. Even if there are those who profess to be able to contemplate with equanimity the sufferings from venereal diseases in a vicious person, surely none, he urged, could remain unmoved at the thought of hundreds of thousands of little children, who, instead of being happy laughing infants, are, solely on account of venereal diseases in their parents, miserable, suffering, diseased, deformed, blind, deaf and imbecile. This is no exaggerated picture; such children can be seen in our asylums, poor-law establishments, hospitals, blind schools, and in hundreds of homes throughout the land. Moreover, as one of the effects of the late war, venereal diseases are almost certainly more prevalent now than they have ever been before in this country.

Dr. Fraser's absolute conviction of the efficacy of self-disinfection and his clear examination of the ethical and moral grounds of objection to the proposal that knowledge should be spread of the simple means available for avoiding these loathsome diseases and their ultimate consequences, secured for him a large majority of support at the Council Meeting in April, 1920. Since that date the clearest and most explicit information on the matter has been disseminated to the public on the authority of the Health Committee.

Experience has already shown that the adoption of this policy has led in Portsmouth to a marked decrease in the number of new cases of disease (there was a drop of practically one-third in 1921), while the few failures justify the warning, stressed in the notices issued by the Council, that the only certain method of avoiding the disease is to lead a chaste life. Of the records of 172 cases carefully analysed in 1921, it is placed on record that 75 men contracted disease in spite of some attempts at disinfection. But in only 15 cases were the conditions of immediate self-disinfection complied with, and in 7 only of these was the advice followed as to the use of potassium permanganate. The experience in 1922 has been somewhat similar. It is obvious that no information is available in regard to those cases in which self-disinfection has apparently resulted in the prevention of disease. Considerable attention has been drawn throughout the country to the action taken in Portsmouth, and to the progress of venereal disease as shown by simple statistics such as those given in the foregoing paragraphs.

It may be added that Portsmouth claims to have been the first area outside London to provide a venereal treatment centre.

TUBERCULOSIS.

Similarly, in 1911, Portsmouth was the first in the field with a dispensary for the supply of tuberculin. Notwithstanding that Dr. Fraser has felt unable to advise his Council to provide a municipal sanatorium, it is noteworthy that the death-rate from pulmonary tuberculosis has shown a substantial decline owing to the general improvement in the conditions of life in the town. Dr. Fraser is not opposed to sanatorium treatment on the ground that it does no good; in many cases, undoubtedly, good, and much good, is effected. What he maintains, however, is that the results so far obtained do not warrant the amount of money that is expended upon them.

In general, he says, that the work carried out for the cure of persons suffering from tuberculosis remains disappointing; this is true whether the treatment be at sanatoria, or at labour colonies, or by tuberculin, or by any of the other methods which at different times seem to promise success. Especially frequent are the disappointments amongst those who have to work hard for their living; so many of this class, after apparently securing considerable improvement from treatment, immediately relapse after returning to their ordinary conditions of life. One great difficulty experienced is that there seems no opening for patients to carry on part-time work of a suitable character at a remunerative wage. If the eradication of tuberculosis is to be effected, it will be accomplished by methods of prevention rather than by those of treatment. At the present time the great overcrowding, due to scarcity of housing accommodation, will, it is feared, very seriously affect the tuberculosis rate in this as in other large towns.

Surgical cases of tuberculosis in children are sent for treatment to the world-famous institution that Sir Wm. Treloar has established at Alton, in Hampshire, and it is also pleasing to learn that the results secured at Beach Lodge, the house in the Langstone Hospital Grounds, set apart as a home for pre-tuberculosis children, have been very satisfactory; children are particularly responsive to improved hygienic conditions, and, almost without any exception, all the patients here show wonderful improvement in their health after a very short time.

HOUSING.

"There are a large number of houses in the borough which are not fit for human habitation regarded from a modern health standard, but under the present conditions of shortage of houses it is inexpedient to close them."

This statement is taken from the Report of the Medical Officer for 1921, and as long as it holds good it cannot but be a source of grave concern to the committee responsible for the public health in Portsmouth. Mr. Childe and his committee are fully alive to the needs of the situation. Between 500 and 600 houses have in fact been erected, of which about 100 occupy the new site at the foot of Portsdown Hill. But this is a long way short of the 2,000 houses which is probably the minimum requirement.

The great value of the Portsdown Hill land to the borough has been continuously urged as a site on which to develop healthy housing accommodation, and it is hoped before long that when by means of a new road access to the town is provided, this site may be completely developed on approved town-planning lines. Mr. Childe pointed out that complaints made in advance that the site is too far out for workmen to live are not worth serious consideration, and seem to ignore the facilities for transport which did not exist a generation ago. The committee propose to go ahead with a scheme for the development of this fine site, and to make a serious effort to get the private builder to overtake the arrears of building. He also stressed the fact that by far the most important feature of the building schemes already undertaken by the Corporation is that there is no overcrowding of houses; 18 houses to the acre in the old borough and 14 to the acre at Portsdown is the maximum. This is a striking improvement on the 40 or more houses to the acre in many parts of the borough. These new houses can never form overcrowded insanitary areas. If, said Mr. Childe, we are to maintain and carry on this idea of doing away once for all with the perpetuation of these overcrowded and insanitary conditions of living for the poorer classes of the community, a hygienic, economic, and social problem second to none facing the country to-day, it is to the periphery we must go, where land is cheap, and building on these lines can become therefore a business proposition. Healthy competition at the periphery will also obviously bring down the enormous price of land in the more central parts of the borough, and so enable the same conditions to be fulfilled in future building in these areas.

Dr. Mearns Fraser expresses appreciation of the support which he has received throughout twenty-six years' service from Mr. Childe and his predecessors and the members of the Public Heath Committees. Abundant evidence was before us both at the interview and in reports and papers which we have since been privileged to read, that if Portsmouth will continue to lend ear to their Medical Officer they will have no difficulty in maintaining their favourable record of health progress. Let us hope that when Mr. Childe welcomes his colleagues of the British Medical Association on their visit next summer he will have the satisfaction of announcing that in the solving of the housing problem Portsmouth is showing the excellent way.

